# Benefits of Endogenous Spatial Attention During Visual Double-Training in Cortically-Blinded Fields

**DOI:** 10.3389/fnins.2022.771623

**Published:** 2022-04-14

**Authors:** Matthew R. Cavanaugh, Duje Tadin, Marisa Carrasco, Krystel R. Huxlin

**Affiliations:** ^1^Flaum Eye Institute and Center for Visual Science, University of Rochester, Rochester, NY, United States; ^2^Department of Brain and Cognitive Sciences and Center for Visual Science, University of Rochester, Rochester, NY, United States; ^3^Department of Psychology and Center for Neural Science, New York University, New York, NY, United States

**Keywords:** hemianopia, rehabilitation, stroke, perceptual learning, motion perception, covert spatial attention, cortical blindness

## Abstract

Recovery of visual discrimination thresholds inside cortically-blinded (CB) fields is most commonly attained at a single, trained location at a time, with iterative progress deeper into the blind field as performance improves over several months. As such, training is slow, inefficient, burdensome, and often frustrating for patients. Here, we investigated whether double-location training, coupled with a covert spatial-attention (SA) pre-cue, could improve the efficiency of training. Nine CB participants completed a randomized, training assignment with either a spatial attention or neutral pre-cue. All trained for a similar length of time on a fine direction discrimination task at two blind field locations simultaneously. Training stimuli and tasks for both cohorts were identical, save for the presence of a central pre-cue, to manipulate endogenous (voluntary) SA, or a Neutral pre-cue. Participants in the SA training cohort demonstrated marked improvements in direction discrimination thresholds, albeit not to normal/intact-field levels; participants in the Neutral training cohort remained impaired. Thus, double-training within cortically blind fields, when coupled with SA pre-cues can significantly improve direction discrimination thresholds at two locations simultaneously, offering a new method to improve performance and reduce the training burden for CB patients. Double-training without SA pre-cues revealed a hitherto unrecognized limitation of cortically-blind visual systems’ ability to improve while processing two stimuli simultaneously. These data could potentially explain why exposure to the typically complex visual environments encountered in everyday life is insufficient to induce visual recovery in CB patients. It is hoped that these new insights will direct both research and therapeutic developments toward methods that can attain better, faster recovery of vision in CB fields.

## Introduction

Unilateral damage to the primary visual cortex (V1) induces vision loss that presents similarly through both eyes, ranging in size from a small scotoma to an entire hemifield (Holmes, 1918; Zhang et al., 2006). This type of vision loss, known as cortically-induced blindness (CB), decreases quality of life (Gall et al., 2009, 2010), causing problems with driving (Parker et al., 2011; De Haan et al., 2014), reading (Leff et al., 2000), and independent living (Chen et al., 2009). In spite of this, only a small percentage of CB patients have access to compensatory therapies, such as eye movement training (Zihl, 1995; Sahraie et al., 2020) or prism lenses (Peli, 2000), and even fewer have access to interventions designed to restore their lost vision (Pollock et al., 2019). Instead, they are usually told to expect some modicum of spontaneous visual recovery in the first few months after their stroke. This is believed to be followed by stabilization of the visual field deficit once patients reach the chronic phase –6 months post stroke– with severely restricted, largely unconscious visual processing abilities remaining inside their blind field (Sanders et al., 1974; Weiskrantz, 1996).

However, experimental work over the last few decades has shown that residual visual processing abilities can be expanded in chronic CB fields, even reaching conscious perception, through the use of visual training approaches (Melnick et al., 2016; Saionz et al., 2020a). Such training, which usually involves repeated visual discrimination or detection of stimuli presented in the blind field, can partially recover motion discrimination (Huxlin et al., 2009; Das et al., 2014; Cavanaugh et al., 2015, 2019), letter identification (Raninen et al., 2007; Chokron et al., 2008), flicker sensitivity (Raninen et al., 2007), luminance and flicker detection (Kasten and Sabel, 1995; Sahraie et al., 2006; Casco et al., 2018), and static orientation discrimination (Das et al., 2014) at the trained, blind-field locations. However, chronic stroke patients typically require thousands of trials over the span of several months to improve performance at a single, blind-field location (Cavanaugh and Huxlin, 2017). Once recovery on a trained task is attained, the stimulus is typically shifted by 1 or more degrees to an adjacent blind-field location, and the entire process begins anew (Huxlin et al., 2009; Cavanaugh and Huxlin, 2017; Saionz et al., 2020b). This successive training design can be effective, but it is not efficient.

Spatial specificity, the inability of training-induced improvements to transfer to untrained locations in the visual field, is a well-documented phenomenon of visual perceptual learning in both visually intact (Jeter et al., 2010; Hung and Seitz, 2014; Donovan et al., 2015) and chronic CB (Sahraie et al., 2006; Huxlin et al., 2009; Cavanaugh and Huxlin, 2017; Saionz et al., 2020b) participants. The difficulty of the task (Ahissar and Hochstein, 1997; Liu and Weinshall, 2000), task precision (Jeter et al., 2009), and the length of training (Jeter et al., 2010; Hung and Seitz, 2014) have been shown to contribute to spatial specificity in intact participants, and have motivated investigations into techniques that could overcome this limitation. In visually intact humans, successful approaches included pre-testing training locations (Zhang et al., 2010), lengthening the time of training (Larcombe et al., 2017), double-location training (Xiao et al., 2008; Mastropasqua et al., 2015; Xie and Yu, 2017), as well as recruiting spatial attention (Donovan et al., 2015, 2020; Donovan and Carrasco, 2018) and feature-based attention (FBA) (Hung and Carrasco, 2021). Some of these techniques have been tested in chronic CB but they did not seem to induce spatial transfer of learning. For instance, patients are typically pre-tested at multiple blind-field locations during baseline mapping of the deficit and to select training locations (Huxlin et al., 2009; Cavanaugh and Huxlin, 2017; Saionz et al., 2020b). Additionally, standard daily training regimens typically extend over several months (Huxlin et al., 2009; Cavanaugh and Huxlin, 2017; Saionz et al., 2020b), and a version of double-training in CB was shown to be effective at inducing transfer between *trained* locations, but not to untrained locations (Das et al., 2014). However, this training was performed using a block design, with one block of 300 trials at one location, a rest period, and a separate block of 300 trials at a second location. Finally, patients trained with FBA were able to recover normal fine direction discrimination thresholds, but improvements were only tested at the single, trained, blind-field locations (Cavanaugh et al., 2019). All in all, for recovery of discrimination thresholds to be attained in cortically-blinded fields using standard, single-stimulus training protocols, the training burden remains high. As a direct reference for the present study, CB patients performed an average (±SD) of 83 ± 24 training sessions (24,840 ± 7,170 trials) to attain stably improved, direction discrimination thresholds at a single, blind-field location (Cavanaugh et al., 2019). Final direction difference thresholds stabilized at an average 9.4 ± 7°, which was approximately 1.5-fold above normal (Cavanaugh et al., 2019). Our task here was to investigate whether it is possible to improve upon this outcome.

Selective visual attention, which prioritizes a subset of sensory information for enhanced processing is thought to play a critical role in visual perceptual learning (for reviews, see Ahissar and Hochstein, 2004; Li et al., 2004; Roelfsema and van Ooyen, 2005; Seitz and Watanabe, 2005; Tsushima and Watanabe, 2009; Watanabe and Sasaki, 2015). Visual spatial attention can be covertly deployed (i.e., without accompanying eye movements) endogenously (voluntarily) or exogenously (involuntarily). Both types of covert spatial attention improve performance on a variety of tasks mediated by early visual processes in visually intact humans (for reviews, see Carrasco, 2011; Carrasco, 2014; Carrasco and Barbot, 2015). Because spatial attention serves as one of the most important mechanisms for gating what and how efficiently information is processed, it is important to investigate how it modulates perceptual learning. However, few studies have systematically manipulated attention to examine this effect in visually intact humans and none in CB. Particularly relevant are studies in which covert spatial attention was manipulated and found to benefit perceptual performance by enabling learning (Szpiro and Carrasco, 2015) and facilitating transfer across locations (Mukai et al., 2011; Donovan et al., 2015, 2020; Szpiro and Carrasco, 2015; Donovan and Carrasco, 2018; Roberts and Carrasco, 2022).

CB patients are also known to deploy, and benefit from, spatial attention within their cortically-blinded fields, despite a lack of awareness in these regions (Pedersini et al., 2020). Here, we asked if double-training CB patients on a direction discrimination task performed with respect to either one of two simultaneously presented visual stimuli on each trial, can restore discrimination performance at *both* blind field locations, and whether covert endogenous spatial attention (SA) during training can facilitate such restoration. Because SA aids visual performance by increasing gain in population responses to visual stimuli (Ling et al., 2009; Barbot et al., 2014; Fernández et al., 2021), it could help overcome spatial specificity in V1-damaged humans, as the blind field of CB patients suffers from both decreased number of neurons and increased internal noise (Cavanaugh et al., 2015). Thus, we posit that double-training along the blind field border, coupled with directing endogenous SA covertly, offers potentially the best chance of recovering function at different locations simultaneously and helping overcome spatial specificity in CB patients. Surpassing this limitation could significantly reduce the amount of training needed to attain recovery at multiple, blind-field locations. This, in turn, would decrease the burden of training, making visual recovery easier to attain.

## Materials and Methods

### Participants

**FIGURE 1:**
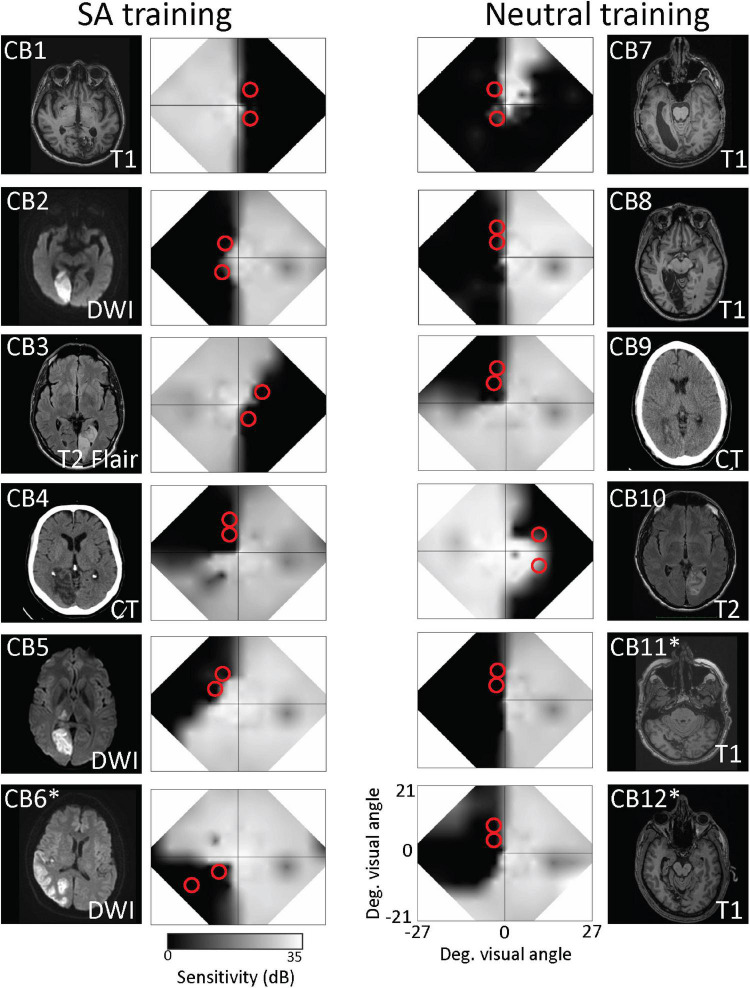
Brain lesions and visual field characteristics of recruited participants. Structural brain scans show locations of stroke-induced damage in the occipital cortex in horizontal sections. Right and left-brain hemispheres are shown according to radiological standards (right brain on image left). Imaging type [MRI T1, diffusion-weighted imaging (DWI), T2-weighted fluid-attenuated inversion recovery (T2-FLAIR), or computed tomography (CT)] is indicated on each image. Patients who did not complete the study are asterisked. Adjacent to each brain image is a composite, binocular map of the central visual field generated from monocular 24–2 and 10–2 Humphrey automated perimetry at baseline. Red circles on each map denote the approximate locations and size of training stimuli.

Data were collected from 12 chronic occipital stroke patients (Table 1). All were at least five months post-occipital stroke (Table 1) affecting the primary visual cortex or its afferent white matter, with resulting visual field deficits measured with standard automated Humphrey perimetry (Figure 1).

**TABLE 1:** Patient demographics and training assignments.

Patient ID	Age	Gender	Time since stroke (months)	Deficit side	Training type	Target locations (x, y) center coordinates	Visit 1–2 interval (months)	# Training sessions at target locations	Completed study?
CB1	62	F	17.1	R	SA	(4, 5)/(4, –5)	2.9	80	Yes
CB2	78	M	6.0	L	SA	(–5, 5)/(–7, –5)	16.6	85	Yes
CB3	50	M	40.1	R	SA	(7, 5)/(3, –5)	12.1	266	Yes
CB4	73	M	10.2	L	SA	(–3, 10)/(–3, 5)	18.7	188	Yes
CB5	26	F	18.1	L	SA	(–8, 5)/(–5, 10)	13.0	143	Yes
CB6	61	M	5.7	L	SA	(–7, –5)/(–16, –10)	N/A	N/A	No
**Mean ± STD**	58.3 ± 18.6		16.2 ± 12.9				12.7 ± 6.1	152 ± 77	
CB7	21	M	48.9	Bilateral	Neutral	(–4, 5)/(–3, –5)	16.2	113	Yes
CB8	51	F	52.2	L	Neutral	(–4, 10)/(–4, 5)	7.1	67	Yes
CB9	51	M	13.1	L	Neutral	(–4, 10)/(–5, 5)	8.0	69	Yes
CB10	49	M	6.0	R	Neutral	(11, 5)/(11, –5)	4.0	76	Yes
CB11	71	M	6.8	L	Neutral	(–3, 10)/(–4, 5)	N/A	N/A	No
CB12	53	M	34.1	L	Neutral	(–4, 10)/(–4, 5)	N/A	N/A	No
**Mean ± STD**	49.3 ± 16.1		26.9 ± 21.0				8.8 ± 5.2	81 ± 21	

*Age and time since stroke are calculated for the date of enrollment. Shaded cells indicate patients who failed to complete their training and return for post-training tests.*

**FIGURE 2:**
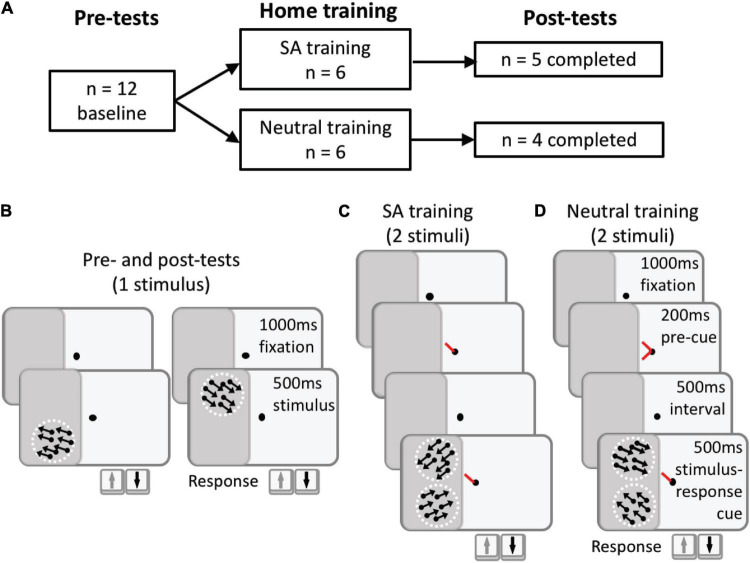
Study and task design. **(A)** 12 Participants were recruited and split between 2 training cohorts. All participants were tested in-lab at baseline, then trained at home for several months, before returning to the lab for post-training measures. Two participants in the Neutral training and 1 participant in the SA training cohorts did not return and were lost to follow up. **(B)** In-lab tests used random dot stimuli moving coherently in a direction above or below the horizontal meridian, either to the left or right, to measure direction difference thresholds relative to the horizontal. Participants were only asked to report if motion direction was above or below the horizontal by pressing the up/down arrows on their keyboard, respectively. All testing was performed with eye-tracker enforced fixation control. **(C)** SA training was identical to the testing task, but additionally included a pre-cue at fixation (represented in red here, white in reality). The pre-cue lasted 200 ms, and was presented 500 ms prior to onset of the 2 stimuli. The SA pre-cue indicated the relative location of an upcoming target stimulus, which would appear in the blind field together with a second, non-target stimulus. The target stimulus, whose direction of motion (above or below the horizontal) the participant was required to indicate *via* key press, was denoted by a response cue presented simultaneously with the 2 stimuli. **(D)** Task sequence for Neutral training, which was identical to SA training, except that the pre-cue (red in diagram) did not indicate which of the 2 upcoming stimuli would be the target.

Participants were excluded if they were unable to fixate properly during perimetry or baseline psychophysical testing performed with an eye tracker. They were also excluded if they presented with any ocular or neurological conditions that could interfere with visual training or testing, including neglect. None of the participants currently used psychoactive drugs, such as anti-depressants, and all had their visual acuity corrected to normal (with glasses or contact lenses) during training and testing. Participants were randomized into 2 double-stimulus/location training cohorts. Nine participants completed training (Figure 2A) and returned to the laboratory for post-training tests and verification of training effects using eye-tracker enforced fixation (see below). Three participants (Table 1, CB6, CB11, and CB12) did not complete their training and declined to return for post-training testing. These participants were thus removed from subsequent analyses. All procedures in the present study were approved by the Institutional Review Board of the University of Rochester Medical Center and adhered to the tenets of the Declaration of Helsinki. Participants were enrolled after giving written, informed consent for participation in the study.

### In-Lab Apparatus and Eye Tracking

Pre- and post-training, in-lab tests were performed with fixation enforced using an Eyelink1000 eye tracker (SR Research, Mississauga, Ontario, Canada). Tracking was binocular for all participants except for two, who suffered from convergence insufficiency (CB7 and CB9); in these two, we tracked the dominant eye and patched the non-dominant eye for all testing and training.

During each trial, participants were asked to fixate on a round, black, fixation target, 0.25° radius, at the center of a mid-gray level CRT monitor (HP 7217A, 48.5 × 31.5 cm, 1,024 × 640 pixel resolution, 120 Hz frame rate), whose luminance was calibrated with a ColorCal MKII automatic calibration system (Cambridge Research Systems, Rochester, United Kingdom). Stimuli appeared in either intact or blind regions of the visual field and were rendered using MATLAB (The MathWorks, Natick, MA) and Psychtoolbox (Brainard, 1997; Pelli, 1997). Viewing distance to the CRT monitor was 42 cm, enforced by a chin/forehead rest. The Eyelink1000 eye tracker is reported accurate by the manufacturer to within 0.25°, with a sampling frequency of 1,000 Hz. During testing, we allowed participants an electronic window of ±1° from the center of the fixation spot. Breaking fixation by moving the eyes outside this window resulted in the trial being aborted, removed from the session and a new trial was generated to replace it.

### In-Lab Psychophysical Testing Before and After Training

Direction difference (DD) thresholds were measured at select, blind-field locations and equivalent locations in the intact hemifield of each participant, generating an internal control for normal performance. The task was a 2-alternative, forced-choice, direction discrimination, in which participants indicated whether global stimulus motion was in a direction angled above or below the horizontal. The trial sequence (Figure 2B) started with stable fixation on the centrally presented fixation spot for 1,000 ms, after which a random-dot stimulus appeared for 500 ms, consisting of black dots on a mid-gray background. Dot density was 3.5 dots per square degree, within a stimulus aperture 5° in diameter, with individual dot lifetime of 250 ms and dot radius of 14 arcmin. Dots moved coherently at 5°/s in one of two base directions (left or right) but at an angle above or below the horizontal. Participants were asked to discriminate if the angle of motion was above or below the horizontal, irrespective of the left/right component of motion. This task-irrelevant information was included to increase the feature-uncertainty between the two stimuli. This reduced the possibility that a participant would base their decision for a target location on motion at the non-target location. Base direction (left/right) and test direction (up/down) were randomly assigned on each trial and for each target. Task difficulty was adjusted on a 3:1 staircase by decreasing the angle above or below the horizontal meridian using the following staircase: 90, 75, 60, 45, 30, 25, 15, 10, 5, 2.5, and 1° difference. DD thresholds were then calculated by fitting performance from a testing session using a Weibull function with a threshold criterion of 72.5% correct (half way between chance performance –50% correct- and 95% correct, which assumed a 5% lapse rate). When performance was too poor (<72.5% correct overall), a nominal threshold value of 90° was assigned to that session. This DD task was similar to our previously described (Cavanaugh et al., 2019) fine direction discrimination task; however, the present task was performed without the feature pre-cues.

To identify suitable blind-field locations for home-training, we first mapped baseline performance at multiple locations across the blind-field border, starting with the 5° diameter random dot stimulus centered on the vertical meridian. The stimulus was moved in 1° lateral increments at a given elevation relative to fixation, and fine discrimination performance (without pre-cues or response cues, Figure 2B) was assessed at each site. Training locations were selected as the first site where DD thresholds were worse than those measured at a roughly corresponding location in the same person’s intact field over a single, 100-trial block, after a 1° lateral movement toward the blind-field (estimated from Humphrey perimetry). Performance at overlapping locations 1–2° deeper into the blind field (*via* lateral shift) was also measured using 100-trials blocks of the same task, to verify that failure persisted distal to the identified training location. The only participant who was an exception to this pattern was CB6, who had been trained as a subacute (CB4 in Saionz et al., 2020b) prior to enrollment in the present study. CB6 exhibited massive transfer of learning deep into their blind field and we could not find a failure point on the DD task until very deep into the blind field during baseline testing for the present study. In addition, this participant was ultimately removed from analyses due to a failure to complete his return visit to the lab to be tested with eye-tracking.

Two potential training locations were selected in each participant (red circles in Figure 1), always at different elevations, at least 5° apart, to prevent potential overlap of the training stimuli. Mean eccentricity of these locations for the 9 patients who completed training was 8.4 ± 2.4° (range: 6–13°), with no significant difference between training cohorts (unpaired *t*-test, equal variance, *t*_16_ = 1.05, *p* = 0.31). Importantly, there was no significant effect of stimulus eccentricity on pre-training/baseline DD thresholds either among the selected blind-field training locations (linear regression: *t*_16_ = 1.22, *r*^2^ = 0.085, *p* = 0.24), or among intact-field, control locations (linear regression: *t*_7_ = –0.76, *r*^2^ = 0.077, *p* = 0.47; Supplementary Figure 1A).

Participants were pseudo-randomized into 2 groups: 6 trained with an SA pre-cue (Figure 2C) and 6 trained with a Neutral pre-cue (Figure 2D); neither the participants nor the researchers were blinded to training assignment. All participants trained at home (see below for description). Of the 6 participants included in the SA training group, 5 completed training and returned to the lab for post-tests (Table 1). Of the 6 participants included in the Neutral training group, 4 completed training and returned to the lab for post-tests (Table 1). The in-lab post-tests were critical to validate training effects with eye-tracker-enforced fixation control. If participants could not return, their data were not included in this study report because we were unable to measure discrimination performance end-points with eye-tracking and without pre-cues at the two trained locations. Post-training tests in-lab were identical to those conducted at baseline.

### Double-Stimulus Training Tasks

The SA training task (Figure 2C) was a modification of the DD task described above for baseline testing (illustrated in Figure 2B). Each trial began with fixation of a small, central target for 1,000 ms, followed by presentation of a pre-cue in the form of a white line extending from fixation, pointing toward one of two possible stimulus locations in the participant’s blind field. The pre-cue was valid and lasted 200 ms, followed by a 500 ms interval. This timing enabled participants to deploy endogenous covert spatial attention specifically to that location ahead of stimulus presentation there. Two random dot stimuli, identical to those used during baseline testing, were then presented simultaneously at the two training locations, concurrent with a response cue identical to the SA pre-cue (i.e., consisting of a single white line at fixation) pointing to one of the 2 stimuli, whose global motion direction relative to horizontal the participant was asked to indicate. The global motion direction of each stimulus was randomized on each trial. Both the stimuli and response cue were presented for 500 ms, after which the participant was allowed to respond by pressing either the up or down arrow keys on their keyboard, to indicate if the perceived global direction of motion at the target location was above or below the horizontal. Auditory feedback was provided following each response to indicate correctness, and the next trial would begin after a 1s inter-trial interval.

The Neutral training task (Figure 2D) was identical to the SA training task, except that the pre-cue consisted of two white lines pointing to both stimulus locations. As such, participants did not know which stimulus would be the target until the response cue was presented simultaneous with the two stimuli.

### At-Home Training Procedures

Training was conducted at home, on participants’ personal computers. Each person was provided with a training program customized to their training locations and their computer specifics (operating system, monitor dimensions, resolution and refresh rate). Training was conducted at a viewing distance of 42 cm, enforced with a lab-issued chinrest (11.5″ Medium Duty, Richmond Products, Albuquerque NM). Participants were instructed that poor fixation during training would inhibit potential visual recovery, and that performance at home would be verified in the laboratory with eye-tracking. They were asked to train for 1 session of 300 trials per day on the direction discrimination task with pre-cues and response cues, 5–7 days per week. At the end of each training session, the program automatically closed and generated a training log of trial-by-trial parameters that was stored on the participant’s computer. The program also created a pop-up at the end of each session, showing the participant their general performance for the session (percent correct and linear threshold). Participants emailed their training logs to the laboratory for analysis and quality monitoring once per week. Compliance with training was not perfect, with a range of training sessions in both cohorts (see Table 1). We aimed for a similar number of training sessions at the blind-field locations of interest before scheduling people to return for in-lab eye-tracker-enforced performance verification, but the amount of time elapsed until the return visit varied significantly. It was affected by the individuals’ rate of improvement, their work/family schedules and ability to travel to our single study site (participants originated from across the entire United States and Canada).

### Data Analysis

Primary outcome measures were change in DD thresholds and the change in % correct performance at minimum difficulty (i.e., 90° DD; Mean Difficulty Performance—MDP). Analyses were performed using MATLAB. Training locations were treated as independent, due to the non-uniform nature of the hemianopic visual field, both in terms of baseline discrimination performance and of training-induced changes in performance (Huxlin et al., 2009; Das et al., 2014; Saionz et al., 2020b). When possible, paired *t*-tests or a Wilcoxon rank sum test were used to compare testing locations and timepoints within a training cohort. However, although two locations were sampled in each person’s blind field, only one intact-field location was tested in the same person. Unpaired *t*-tests were performed because the two blind-field locations behaved independently and created an uneven comparison. *F*-tests were performed to determine if variability was similar between groups, followed by either an equal or unequal-variance, unpaired *t*-test as appropriate. *F*-test values for all comparisons are reported in Supplementary Table 1. Degrees of freedom, *t*-values and *p*-values are reported for all *t*-tests in the text of the “Results” section, and all *t*-tests were two-tailed.

## Results

### Baseline Fine Direction Difference Performance

**FIGURE 3:**
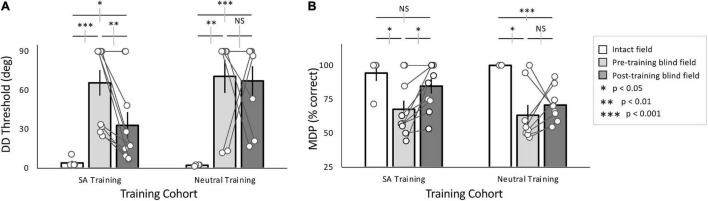
Effects of training with SA- and Neutral pre-cues. **(A)** Plot of mean (± SEM) direction difference (DD) threshold, with individual data superimposed (white circles). Intact field performance was largely normal across both cohorts (white bars), while baseline (pre-training) performance in the blind field was similarly impaired for both cohorts (light gray bars). After training (dark gray bars), the majority of SA-trained participants, and the group as a whole improved over baseline. However, the group average remained impaired relative to the intact field. In contrast, the majority of Neutral-trained participants did not attain lower thresholds after training. As a group, they did not improve from baseline and remained impaired relative to their intact field. **(B)** Plot of mean (± SEM) percent correct performance at the minimum difficulty (MDP), in this case a 90° DD from horizontal. Intact field performance for both cohorts was similarly good (white bars), and baseline performance in the blind field was impaired (light gray bars) relative to their intact visual fields. All participants in the SA training cohort improved relative to their own pre-training levels, attaining post-training MDP % correct performance that was not significantly different from their intact field. In comparison, the Neutral-trained cohort did not improve significantly from baseline overall, and remained impaired relative to their own intact fields. Gray error bars on comparisons lines indicate ±1 standard error of the difference between the compared means; see text for descriptive statistics.

Baseline performance was collected for all participants in the SA- and Neutral-training cohorts at two, blind-field locations. Participants in the SA-training cohort averaged DD thresholds of 65.8 ± 31.3° and MDP of 67.6 ± 20.1% correct across all blind-field locations (Figures 3A,B). In contrast, in their intact field, DD thresholds averaged 4.1 ± 3.8° and MDP was 94.3 ± 12.8% correct (Figures 3A,B). Blind-field performance was thus significantly impaired at baseline relative to the intact field (Wilcoxon rank sum: Threshold: *p* = 0.0006, MDP: *p* = 0.024).

The Neutral-training cohort had average DD thresholds of 70.6 ± 35.9° and MDP of 63.3 ± 21.3% correct across blind-field training locations (Figures 3A,B), significantly worse than performance in the intact field, averaging 2.2 ± 0.5° and MDP was 100 ± 0% correct (Figures 3A,B; Wilcoxon rank sum: Threshold: *p* = 0.002, MDP: *p* = 0.03).

Overall, baseline performance was similar in the two cohorts both in the blind (Threshold: unpaired *t*-test, equal variance *t*_16_ = –0.30, *p* = 0.77; MDP: unpaired *t*-test, *t*_16_ = 0.44, *p* = 0.66) and intact (Threshold: unpaired *t*-test, *t*_7_ = 0.95, *p* = 0.37; MDP: unpaired *t*-test, *t*_7_ = –0.88, *p* = 0.41) fields. Moreover, eccentricity of the target blind-field locations was not significantly correlated with baseline DD thresholds (linear regression: *t*_16_ = –1.88, *r*^2^ = 0.077, *p* = 0.24; white data points in Supplementary Figure 1A). Importantly, DD thresholds in the intact field also did not vary with eccentricity (linear regression: *t*_7_ = –0.76, *r*^2^ = 0.085, *p* = 0.47; gray data points in Supplementary Figure 1A).

### Impact of Training on Blind-Field Performance

SA-trained participants attained post-training DD thresholds of 33.0 ± 32.4° (Figure 3A) and an MDP of 84.7 ± 16.9% correct (Figure 3B). This performance was significantly better than pre-training measures (paired *t*-tests, DD thresholds: *t*_9_ = 3.43, *p* = 0.0075; MDP: *t*_9_ = –3.25, *p* = 0.010). However, whereas the MDP was now similar between post-training blind-field and intact locations (unpaired *t*-test, equal variance, *t*_13_ = 1.11, *p* = 0.29), DD thresholds failed to reach intact-field levels of performance (unpaired *t*-test, unequal variance: *t*_9.47_ = –2.78, *p* = 0.020).

Participants in the Neutral-trained cohort attained post-training DD thresholds averaging 67.2 ± 32.4°, and MDP was 71 ± 13% correct (Figures 3A,B). Unlike the SA cohort, the Neutral training group exhibited no significant improvements at their trained, blind-field locations (paired *t*-tests, DD thresholds: *t*_7_ = –0.17, *p* = 0.87; MDP: *t*_7_ = –0.86, *p* = 0.42); performance remained impaired relative to the intact field (unpaired *t*-test unequal variance, DD thresholds: *t*_7_ = –5.67, *p* = 0.00076; MDP: *t*_7_ = 6.37, *p* = 0.00038).

In sum, although both groups started with similar baseline performance, SA-trained participants attained significantly better DD thresholds than Neutral-trained participants (unpaired *t*-test, equal variance *t*_16_ = –2.22, *p* = 0.041). Finally, eccentricity of the trained, blind-field locations did not reliably influence training outcomes, with no significant effect of eccentricity on change in DD thresholds (linear regression: *t*_16_ = 1.22, *r*^2^ = 0.182, *p* = 0.08; Supplementary Figure 1B).

### Impact of Baseline Performance on Training Outcomes

**FIGURE 4:**
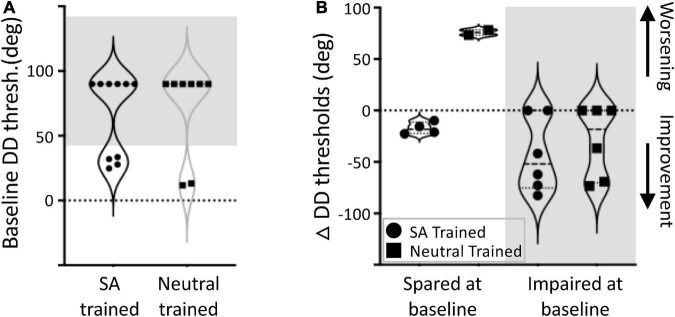
Change in direction difference (DD) thresholds sorted by baseline performance. **(A)** Blind-field training locations were categorized as “impaired” at baseline if DD threshold was > 45° (shaded region), or as relatively “spared” if DD threshold was <45 °. Four SA- and two Neutral-trained participants had a single location each categorized as having “spared” vision. Four SA- and two Neutral-trained participants had a single location each categorized as “impaired” prior to training, whereas one SA and two Neutral-trained participants were impaired at baseline at both of their training locations. **(B)** Spared locations (white region), when SA-trained, maintained their spared performance or slightly improved; when such locations were Neutral-trained, performance became significantly worse. For both training cohorts, when initial performance was impaired (shaded region), improvements were seen in about half of the participants; the rest showed no change.

Although baseline performance was similar in the two training cohorts, there was significant variability among participants, and even between training locations in a given participant. To determine if baseline performance impacted training outcomes, we sorted training locations in a binary manner according to whether they exhibited pre-training DD thresholds above or below 45° (Figure 4). If DD thresholds were < 45°, the location was labeled as “Spared at Baseline.” If DD thresholds were > 45°, the location was labeled “Impaired at Baseline.” Note that being categorized as “Spared” did *not* imply that a given location had normal DD sensitivity, but rather that sensitivity could be measured. Locations were further sorted by training type, across all participants. We found that 6/18 locations had “Spared” vision (4 SA trained, 2 Neutral trained), and the remainder were “Impaired” (6 SA trained, 6 Neutral trained, Figure 4A). The large gap in performance within each of the two groups of participants (Figure 4A) justifies this division into separate groups.

Locations with “Spared” vision that underwent SA-training maintained their spared performance or improved slightly, averaging a DD change of –17.3 ± 5.8° (Figure 4B). In contrast, the two “Spared” locations that underwent Neutral-training became considerably worse post-training, with DD thresholds increasing by 78.2 and 73.3°, respectively. For both training cohorts, when the initial training location was “Impaired” at baseline, 4/6 SA-trained and 3/6 Neutral-trained locations improved—the rest did not change. Correspondingly, a two-way ANOVA revealed a significant effect of training type [*F*_(1_, _17)_ = 11.5, *p* = 0.0045], and a significant effect of baseline performance [*F*_(1_, _17)_ = 17.5, *p* = 0.0009] with a significant interaction effect [*F*_(1_, _17)_ = 6.42, *p* = 0.024].

### Comparing Performance Between the Two Training Locations

**FIGURE 5:**
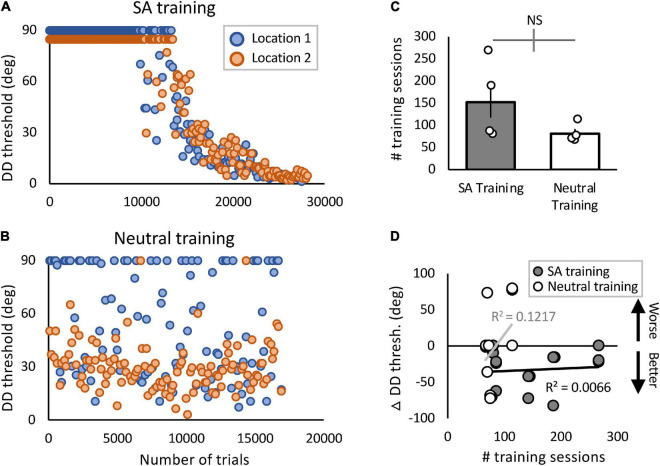
Blind-field performance across training trials and sessions. **(A,B)** Example plots of 2 participants’ thresholds as a function of # trials during home training (A—CB4; B—CB7). Orange and blue data points represent daily performance at the two trained locations in each person, with 150 trials/session/location. In **(B)**, Locations 1 and 2 had the better and worse initial performance, respectively. In **(A)**, Location 2 sessions with a threshold of 90 are presented as 85 for illustrative purposes. **(C)** Plot of the total number of sessions trained in each cohort (means ± SEM) with individual data superimposed. There were no significant differences between groups. Gray error bar on the comparison line indicates ± 1 standard error of the difference between the compared means. **(D)** Plot of pre- to post-training change in DD threshold against the total number of training sessions performed by participants in the two training cohorts. There was no significant correlation between amount of training performed and DD thresholds in either training group (SA: *t*_8_ = 0.23, *p* = 0.82; Neutral: *t*_6_ = 0.91, *p* = 0.40).

A potential concern with the present protocol is that participants may not have split their attention equally between the two stimulated, blind-field locations during training. To address this possibility, we examined progression of home-training performance at both stimulated, blind-field locations for individuals in each training cohort (Figure 5 and Supplementary Figure 2), and found well-matched progression/lack of progression at both locations in each participant, in both cohorts.

However, in addition to the lack of eye-tracker-enforced fixation control, the home-training task differed markedly from that used for in-lab measurements by including both attentional pre-cues and response cues (absent in-lab) and 2 simultaneously presented stimuli (vs. a single stimulus in-lab). Thus, we also examined changes at the two training locations, comparing in-lab pre- and post-training measurements. Training locations were separated into Location 1 and 2, according to baseline performance, with Location 1 displaying better performance at baseline. Note that “worse” performance was not necessarily an inability to perform the task, and “better” performance did not necessarily mean normal thresholds. If baseline performance was similar at the two locations [which occurred in one SA-trained participant (CB5) and two Neutral-trained participants (CB8, CB10)], these were designated “1” or “2” at random. There were no significant differences between locations 1 and 2 in-lab DD thresholds of participants in either the SA cohort or the Neutral cohort (Supplementary Figures 3A,C). This result was also consistent for MDP (Supplementary Figures 3B,D). Thus, both locations had similar changes for both measures.

### Impact of Amount of Training on Behavioral Outcomes

A potential cause for SA-trained participants improving more than Neutral-trained ones could be differences in the amount of training performed. To test this possibility, we compared the total number of trials and sessions performed at home at the specified target locations. On average, SA-group participants trained for a total of 22,860 ± 11,629 (SD) trials per location, whereas Neutral-group participants trained for 12,187 ± 3,227 (SD) trials (see Table 1 for number of sessions trained). The greater average number of sessions and trials performed by the SA-training group was largely driven by one SA-trained participant (CB3), who in spite of repeated reminders to only train once per day, failed to comply. He trained 2–4 sessions per day for 180 days. By the same token, three of the Neutral-trained participants (CB7, 8 and 9) failed to train 5 days per week, and ended up with fewer training sessions than they were asked to perform before returning to the lab for verification (Table 1). In spite of these differences in training rate, a regression analysis showed that the number of sessions/trials trained accounted for only minimal variance of in-lab-measured change in DD thresholds overall (linear regression: *R*^2^ = 0.0094, *t*_16_ = –0.39, *p* = 0.70), and for either training cohort (Figure 5D).

## Discussion

Our primary goal in this study was to assess the efficacy of double-location training, with or without SA pre-cues, to recover visual function and to overcome some of the limitations induced by the intrinsic spatial specificity of visual recovery in CB patients undergoing single-location training. To this end, we combined several established methods of addressing spatial specificity: (1) Presenting two stimuli at different blind-field locations simultaneously, (2) using an endogenous, spatial pre-cue to guide attention prior to stimulus presentation, and (3) using extended training periods. We found that double-training coupled with SA pre-cues could recover DD thresholds at two blind-field locations simultaneously. Moreover, it did so using a comparable number of trials as previously shown to attain stable improvement with single-stimulus training (Cavanaugh et al., 2019). To our knowledge, this is only the second study (Pedersini et al., 2020) to utilize deployment of covert, endogenous SA within cortically-blinded fields, and the first to reveal that this deployment benefits visual training. This result sets the stage for future investigations that manipulate attention to enhance training efficacy and efficiency.

### Spatial Attention Pre-cues Facilitate Double-Training in Cortically-Blinded Fields

Double-location training with SA pre-cues at fixation improved direction discrimination thresholds simultaneously at two blind-field locations and to a similar extent. Spatial attention boosts in gain of the population response (Ling et al., 2009; Barbot et al., 2014; Fernández et al., 2021) aid in situations in which high internal noise is the limiting factor on performance. Hence, the improvement in performance attained by double-location training with SA pre-cues is consistent with our finding that blind-field performance is primarily limited by high internal noise (Cavanaugh et al., 2015).

SA improved performance at both locations, even though deployment of SA to a target location can concurrently impair processing of stimuli presented at unattended locations (Posner et al., 1980; Pestilli and Carrasco, 2005; Rihs et al., 2007; Montagna et al., 2009; Herrmann et al., 2010). In addition, the nature of the double-location training protocol introduced additional spatial uncertainty not present in our single-location protocols. Increased spatial uncertainty impairs performance in both typical and patient populations, but can be overcome by guiding attention (Herrmann et al., 2010; Phu et al., 2018). Thus, the pre-cue and subsequent deployment of SA to target training locations may have boosted performance and training efficacy through a combination of increased gain at the target and reduced processing of the distractor. The efficacy of SA is especially interesting because patients presumably attend to locations within their cortically-blinded field, where they report degraded or completely absent conscious vision.

However, the use of SA pre-cues during double training was not sufficient to improve fine discrimination thresholds to the same level as single-location training with feature pre-cues, which returned DD thresholds to intact field levels (Cavanaugh et al., 2019). A possible mechanism behind this limitation is that SA is thought to improve performance by boosting cell response gain and, unlike FBA, does not enhance tuning (Baldassi and Verghese, 2005; Ling et al., 2009; Barbot et al., 2014; Fernández et al., 2021). A boost in gain would be most beneficial when discriminations are coarse, while enhanced tuning would be most beneficial for fine discriminations, such as in this study. Thus, whereas SA pre-cues during training were able to overcome limitations to performance imposed by increased internal noise within cortically-blinded fields (Cavanaugh et al., 2015), they were less effective for finer discrimination, when internal noise was no longer the limiting factor to performance (Dosher and Lu, 1998). Thus, performance improved but residually-high thresholds persisted. Given that DD thresholds can reach intact field levels of performance following FBA training (Cavanaugh et al., 2019), these limitations are likely not inherent to the visual system. Rather, SA was likely insufficient to overcome all of the hurdles present in a damaged visual system. Future efforts to train CB people with FBA in a double-location training protocol could be informative. If such a paradigm restored thresholds to intact-field levels, it would suggest that the primary limitation in the present training configuration was overly broad tuning of the population response. If not, it would suggest that the damaged visual system is simply not able to effectively divide resources between two, simultaneously presented, blind-field training locations.

### Impact of Baseline Performance and Training Type on Recovery

In our previous studies, all training locations (though limited) were equivalently impaired prior to training, whereas a portion of training locations in this study possessed some residual, though still worse than normal, direction discrimination performance. A coarse assessment of our “spared vs. impaired” classification revealed—for the first time—that locations with residual threshold performance may be differently affected by training than locations that are fully impaired prior to training. Indeed, at least half the impaired locations trained in either group improved. In contrast, most spared locations improved further when trained with the SA task, whereas both spared locations trained on the Neutral task became impaired. Future efforts involving a larger number of participants will be needed to fully establish the impact of baseline vision on training outcomes, both for single and double-location training protocols.

### Limitations of Double-Training Without Spatial-Attention Cues

We were surprised to find that double-location training with Neutral pre-cues failed to improve threshold performance in 3/4 of those tested (5/8 locations). Whereas such training can improve performance in visually intact participants (Zhang et al., 2010; Donovan et al., 2015, 2020; Mastropasqua et al., 2015; Xie and Yu, 2017; Donovan and Carrasco, 2018) the damaged visual system may not appropriately distribute perceptual resources during simultaneous stimulus presentations. The presence of a second stimulus may in fact serve as a distractor when performing the task at the target location. Perhaps this arises from an inability to exclude the secondary location from processing by the already-limited visual system, abnormalities of suppression/inhibition and/or to an expansion of receptive field sizes within the deficit zone (Papanikolaou et al., 2014; Barbot et al., 2021).

Alternatively, our result may be explained by the introduction of spatial uncertainty to the training task in the double-location condition, which is not present in single-location protocols. Visual and attentional resources, already limited by the reduction of visual processing units in the brain, must now be spread across two locations. Another possible explanation is that the presentation of response cues simultaneous with the stimuli did not provide the damaged visual system enough time to process the cue, orient to the location of interest and interpret the target. Determining whether processing times are similar in CB and visually intact controls may allow further optimization of stimulus and task parameters for CB training. Finally, Neutral-trained CB participants could ultimately show performance improvements if allowed to train for much longer than was done here; however, their ability to maintain compliance in the face of extremely slow or no progress remains a challenge for such an experiment.

### Comparison of Amount of Training Needed in Single and Double-Stimulus Protocols

Another important result that emerged from the present experiment is that the number of training sessions or trials performed did not consistently explain differences in training efficacy between SA- and Neutral-trained participants. In addition, these participants trained for a similar length of time and sessions as the participants in our single-location training study (Cavanaugh et al., 2019). However, SA-trained participants were able to recover fairly good discrimination performance at two blind-field locations at once. Thus, the overall training burden was reduced by about half in terms of the number of trials performed relative to single-location training. This finding suggests that multi-location training, when coupled with SA cues, can improve training efficiency. However, speed and reduced effort did not come with best quality. Final DD thresholds attained here (median of ∼18 °) were nowhere near intact-field levels (median of ∼2.5°), nor levels attained with single-stimulus training (Cavanaugh et al., 2019). Notably, participants in this prior study were trained at two separate blind-field locations each day, but in a blocked design (300 trials at one location, followed by another 300 trials at the second location), rather than with randomly alternating trials. That final performance in the 2019 study was better than what was observed in the present study is consistent with the fact that spatial uncertainty can be detrimental as well as with the notion that there may be a limitation to spatial attentional deployment in CB patients.

We do not know which training method and outcome is preferable for patients: coarser discrimination abilities at 2 blind-field locations vs. normal discrimination abilities at a single blind-field location. Unlike other visual impairments (e.g., glaucoma, macular degeneration), in which the magnitude of the impairment correlates almost linearly with a drop in quality of life (Mckean-Cowdin et al., 2007), the size of a CB deficit does not correlate well with quality of life (Papageorgiou et al., 2007; Gall et al., 2010). Instead, the location of the field cut appears more critical (Papageorgiou et al., 2007). However, it must be noted that clinically, measures of visual impairment in this population are often derived from automated luminance detection perimetry, rather than visual discrimination thresholds, making comparisons between our results and the above-mentioned studies difficult. As such, it remains unclear whether patients are better served by partially recovering discrimination thresholds faster, *via* SA training, or normal thresholds slower, *via* FBA training. An option is to design a training protocol that combines both training types, perhaps priming two locations to recover quickly with SA, then restoring normal thresholds with FBA cues. This idea is supported by the finding that with intact vision, FBA training benefits generalize to untrained locations and are long lasting (Hung and Carrasco, 2021).

### Implications for Daily Living

A common question regarding training-induced recovery in CB is why patients do not recover discrimination abilities in their blind-field simply from being exposed to visual stimulation that arises in their day-to-day activities. Instead, extensive, focused training appears necessary to induce even modest improvements in visual performance. Our present findings, that splitting attentional resources between just two locations within a cortically-blind field (in the Neutral-trained protocol) fails to consistently attain recovery at either location, begins to address this question. Damage to the visual system may limit visual resources available for deployment across the visual field, making the limited information processed coarse and noisy. Thus, there may be insufficient gain to evoke perceptual learning and recovery from simple exposure. By directing limited resources toward a single location, either by only presenting single stimuli, or through endogenous SA manipulations, these barriers can be at least partially overcome.

People may thus benefit from training protocols that incorporate attentional cues, or from training designed—*via* pre-cues—to support the deployment of attention sequentially around the visual field (Green and Bavelier, 2006a). It may be tempting to consider that training with complex, naturalistic stimuli and environments, such as those generated using virtual reality or action video games (Green and Bavelier, 2003; Green and Bavelier, 2006a), may be more attractive as training tools than the reduced, simplistic and less entertaining psychophysical approaches used here. If “gamified” training protocols are able to improve working memory and cognitive control (Green and Bavelier, 2006b; Colzato et al., 2012) in CB, they could function to enhance a participant’s ability to recover. However, video game-based training often relies on specific properties of video games, e.g., an ability to deploy attention across the visual field, to quickly process visual information, and to switch between attention modes (Bavelier and Green, 2019). If CB patients, by the nature of their injury, are unable to perform under such conditions, then video game training may not be suitable for this population.

## Conclusion

The present study showed, for the first time, that double-location training coupled with endogenous SA pre-cues is able to improve DD thresholds at two cortically blind locations simultaneously. Whereas this training was not able to restore DD thresholds back to normal, it induced meaningful improvements with a markedly reduced training burden compared to single-location training. Our findings show that attention manipulations can benefit training outcomes in CB patients. They also reveal a possible limitation in this population with respect to their ability to process multiple stimuli simultaneously without spatial cueing. Understanding both the capacity and limitations of visual processing in CB remains key for designing appropriate and maximally effective training protocols.

## Data Availability Statement

The raw data supporting the conclusions of this article will be made available by the authors, without undue reservation.

## Ethics Statement

The studies involving human participants were reviewed and approved by the Institutional Review Board of the University of Rochester Medical Center. The patients/participants provided their written informed consent to participate in this study.

## Author Contributions

MRC, KH, DT, and MC designed the study. MRC performed the experiments and collected the data, wrote the manuscript. MRC, KH, and DT analyzed the data. All authors commented and edited the manuscript and worked on the interpretation of the data.

## Conflict of Interest

The authors declare that the research was conducted in the absence of any commercial or financial relationships that could be construed as a potential conflict of interest.

## Publisher’s Note

All claims expressed in this article are solely those of the authors and do not necessarily represent those of their affiliated organizations, or those of the publisher, the editors and the reviewers. Any product that may be evaluated in this article, or claim that may be made by its manufacturer, is not guaranteed or endorsed by the publisher.
