# Supplementary material for: Benefits of Endogenous Spatial Attention During Visual Double-Training in Cortically-Blinded Fields
Source: Front Neurosci. 2022 Apr 14;16:771623. doi: 10.3389/fnins.2022.771623 (PMC9046589; doi:10.3389/fnins.2022.771623)
Supplement: Supplementary file 1 [file Table_1.DOCX]

**Supplement Table 1: Results from two-sample F-tests for equal variance**. Before running unpaired t-tests, the samples to be compared were first assessed to determine if the t-test should be adjusted for equal or unequal variance. All comparisons in which this analysis was necessary are listed here. The “Test” column indicates which comparison is being assessed, the “DF” column indicates the degrees of freedom for the analysis, the “F value” column provides the F-statistic from the test, and the “P value” column indicates the p-value of the analysis. A p-value of less than 0.05 indicates unequal variance, while greater than 0.05 indicates equal variance. Note that this p-value indicates whether the listed comparison should be made with an equal or unequal variance t-test, and does not indicate the significance of that t-test.

| **Test** | **DF** | **F value** | **P value** |
| --- | --- | --- | --- |
| SA Cohort, Pre-training, Intact field vs Blind field thresholds | 4,9 | 0.014 | 0.00097 |
| SA Cohort, Pre-training, Intact field vs Blind field MDP | 4,9 | 0.4 | 0.4 |
| Neutral Cohort, Pre-training, Intact field vs Blind field thresholds | 3,7 | 0.00022 | 0.00001 |
| Neutral Cohort, Pre-training, Intact field vs Blind field MDP | 3,7 | 0.0 | 0.0 |
| SA Cohort, Pre-training Blind field vs Neutral Cohort, Pre-training Blind field thresholds | 9,7 | 0.76 | 0.69 |
| SA Cohort, Pre-training Blind field vs Neutral Cohort, Pre-training Blind field MDP | 9,7 | 0.89 | 0.85 |
| SA Cohort, Post-training, Intact field vs Blind field thresholds | 4,9 | 0.013 | 0.00085 |
| SA Cohort, Post-training, Intact field vs Blind field MDP | 4,9 | 0.57 | 0.62 |
| Neutral Cohort, Post-training, Intact field vs Blind field thresholds | 3,7 | 0.00027 | 0.00014 |
| Neutral Cohort, Post-training, Intact field vs Blind field MDP | 3,7 | 0.0 | 0.0 |
| SA Cohort, Post-training Blind field vs Neutral Cohort, Post-training Blind field thresholds | 9,7 | 1.00 | 0.98 |
| SA Cohort, Post-training Blind field vs Neutral Cohort, Post-training Blind field MDP | 9,7 | 1.70 | 0.49 |
| SA vs Neutral Cohort number of training trials | 4,3 | 6.23 | 0.16 |
| SA vs Neutral Cohort number of training sessions | 4,3 | 6.23 | 0.16 |
