# Supplementary figures and images for: Benefits of Endogenous Spatial Attention During Visual Double-Training in Cortically-Blinded Fields
Source: Front Neurosci. 2022 Apr 14;16:771623. doi: 10.3389/fnins.2022.771623 (PMC9046589; doi:10.3389/fnins.2022.771623)

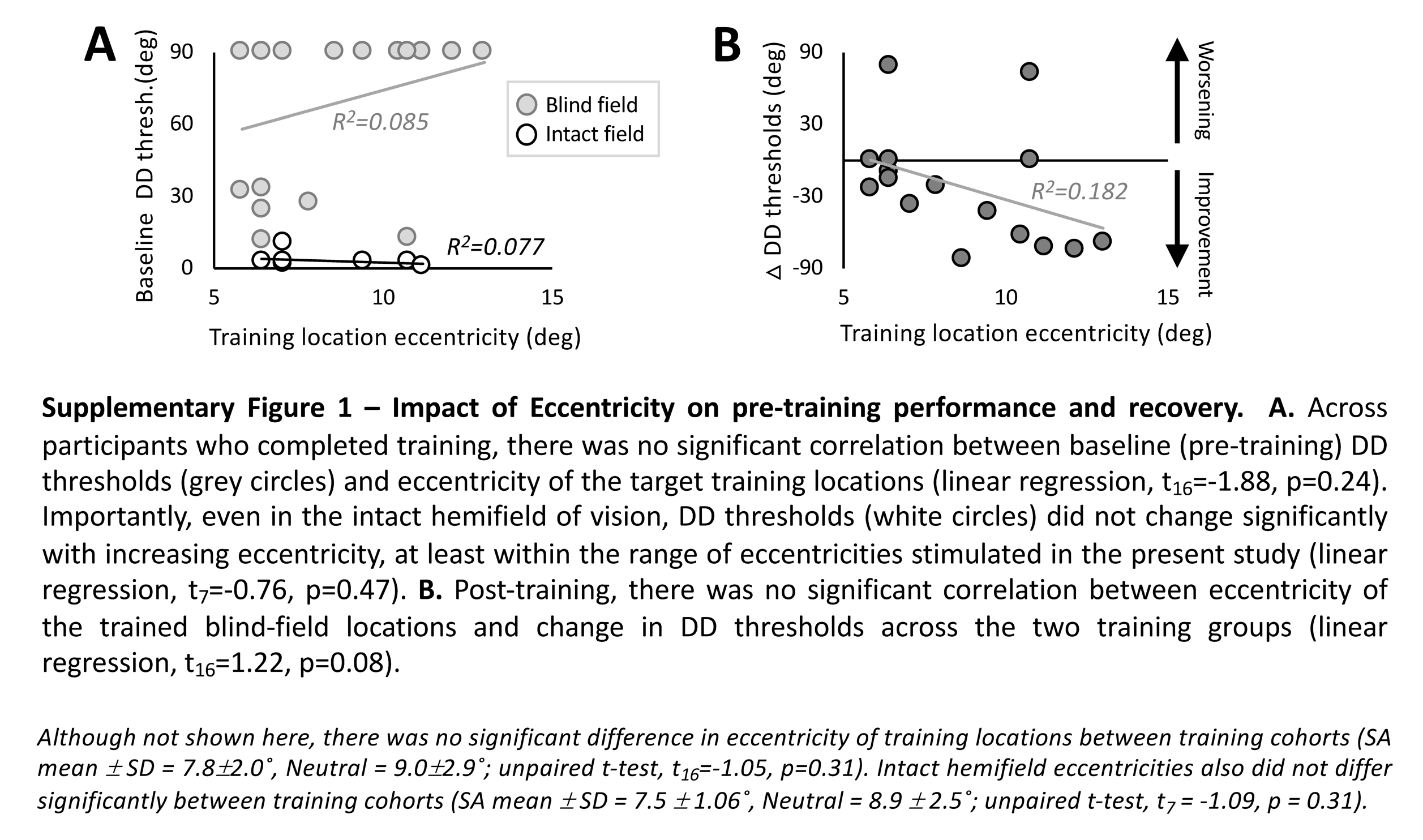

Supplement: Supplementary file 2 [file Image_1.TIFF]
